# Time to Mortality and Predictive Factors Among Adult Heart Failure Patients: Lessons From a Resource-Limited Setting

**DOI:** 10.1155/crp/3968055

**Published:** 2025-08-07

**Authors:** Elsah Tegene Asefa, Tamirat Godebo Woyimo, Hikma Fedlu Bame, Eyob Girma Abera

**Affiliations:** ^1^Department of Internal Medicine, Jimma University, Jimma, Oromia, Ethiopia; ^2^Department of Public Health, Jimma University, Jimma, Oromia, Ethiopia

**Keywords:** Cox regression, Ethiopia, heart failure, Kaplan–Meier estimator, mortality

## Abstract

**Background:** Heart failure (HF) is a major cause of morbidity and mortality in low-resource settings like Ethiopia. This study aimed to assess time to mortality and identify key predictors among adult HF patients at Jimma Medical Center (JMC).

**Methods:** A retrospective cohort study was conducted on 356 adult HF patients admitted to JMC between 2022 and 2023. Survival probabilities were estimated using the Kaplan–Meier method, and Cox proportional hazard regression was used to identify mortality predictors.

**Results:** Among 356 HF patients, 15.7% (95% CI: 12.2%–19.8%) died during the study period. The median hospital stay was 11 days (IQR: 7–17), and the median age was 55 years (IQR: 38–65). Key predictors of higher mortality included hypertension (AHR: 4.6, 95% CI: 1.88–11.61, *p* < 0.001), pneumonia (AHR: 4.3, 95% CI: 1.15–15.78, *p* = 0.031), anemia (AHR: 3.3, 95% CI: 1.17–9.06, *p* = 0.023), acute myocardial infarction (AMI) (AHR: 4.4, 95% CI: 1.9–10.09, *p* < 0.001), and hyponatremia (AHR: 2.9, 95% CI: 1.44–5.99, *p* = 0.003). Each unit increase in systolic blood pressure (SBP) and diastolic blood pressure (DBP) was linked to a 7% and 4% lower mortality risk, respectively (*p* = 0.035). A higher pulse rate was associated with a 4% increased mortality risk. Patients with heart failure with reduced ejection fraction (HFrEF) had a six-fold higher mortality risk compared to those with preserved ejection fraction (HFpEF) (AHR: 6.1, 95% CI: 1.79–24.4, *p* = 0.008).

**Conclusion:** This study identifies key mortality predictors for HF patients in a resource-limited setting, including hypertension, pneumonia, anemia, AMI, and hyponatremia. The findings emphasize the need for targeted interventions, improved management strategies, and policies to reduce HF mortality in low-resource environments. Further research is needed to refine these findings and enhance care for HF patients in such settings.

## 1. Introduction

Heart failure (HF) is a multifaceted clinical syndrome characterized by the heart's impaired ability to pump blood effectively, leading to inadequate perfusion of tissues and organs [1]. Globally, HF is a significant public health issue, with escalating prevalence and high rates of morbidity and mortality. According to the Global Burden of Disease Study, HF contributes to over 2 million deaths each year, underscoring its critical impact on global health [2].

Mortality rates among patients with HF are still high, yet the specific causes of death are often unclear [3]. Although the risk of death is significant for those with HF, many forms of HF can be prevented through a healthy lifestyle [4]. Once HF is present, early medical intervention can help prevent premature deaths. Additionally, many patients with HF have other health conditions that significantly impact their overall health experience [5, 6].

In Ethiopia, cardiovascular diseases, including HF, are emerging as major health concerns. The prevalence of HF in Ethiopia has been increasing over recent decades, reflecting broader trends seen across low- and middle-income countries (LMICs) [7]. This rising burden of HF has profound implications for patients, families, and the healthcare system, exacerbating the need for effective management strategies [8, 9].

Despite the growing prevalence of HF in Ethiopia, research on factors influencing mortality among HF patients within the country remains limited. Studies conducted in LMICs have identified various predictors of HF mortality, including demographic factors, comorbidities, clinical presentation, and treatment modalities [10]. However, there is a notable scarcity of data specific to Ethiopian populations, which hinders the development of localized, evidence-based treatment guidelines.

Understanding the time to mortality and predicting factors among adult HF patients is essential for improving patient outcomes and optimizing healthcare resources. The findings of this study may have significant clinical implications by identifying high-risk patients who require closer monitoring and targeted interventions. This, in turn, may contribute to reducing mortality rates and improving the quality of life for HF patients. Furthermore, the results can guide healthcare policymakers and stakeholders in resource allocation and planning for the management of HF at both the individual and population levels.

Therefore, this study, conducted at Jimma Medical Center (JMC), aims to address this gap by examining time to mortality and identifying predictive factors among adult HF patients. By analyzing clinical data from a resource-limited setting, we aimed to provide insights that can inform targeted interventions and improve patient outcomes in Ethiopia and similar contexts.

## 2. Methods

### 2.1. Study Design and Setting

A hospital-based retrospective cohort study was conducted using 356 inpatient files from JMC for the years 2022 and 2023. The hospital, located in Jimma town, 350 km southwest of Addis Ababa, offers specialized healthcare services through its nine medical departments.

### 2.2. Eligibility Criteria

Adult patients (aged 18 years and above) who were diagnosed with HF and admitted to JMC between 2022 and 2023 were included. Patients with incomplete medical records or missing key variables required for analysis were excluded from the study.

### 2.3. Data Collection

Patient data were collected from April 15 to May 14, 2024. Patient sociodemographic (age, sex, and residence) and clinical characteristics (symptom profiles, vital signs, medical conditions, and HF-specific data such as etiology, types, stages, and classification) were collected from patient files using checklists developed specifically for this study. Data collection was performed by trained research assistants following a standardized data collection form.

### 2.4. Data Management and Statistical Analysis

The original data collected in Microsoft Excel were reviewed for completeness and consistency before being exported to SPSS® Version 26 (IBM®, New York, USA) for analysis. Normality tests were conducted using visual inspections of histograms and Q-Q plots, as well as the Kolmogorov–Smirnov and Shapiro–Wilk tests. For categorical variables, associations with mortality were assessed using the chi-squared test or Fisher's exact test when the expected cell count was less than five. For continuous variables that were not normally distributed, nonparametric tests were applied. The Kaplan–Meier estimator, along with equality tests such as the log-rank (Mantel–Cox), Breslow (generalized Wilcoxon), and Tarone–Ware tests, was used to estimate survival curves for categorical predictors, with patients who experienced death during the observation period being the event of interest. Additionally, univariate Cox regression analyses were conducted to assess individual factors associated with the hazard of death among adult HF patients. All variables with a *p* ≤ 0.25 in the bivariable analysis were included in a multivariable analysis. Variables with fewer than five occurrences were excluded due to insufficient sample size for reliable estimates. In the final model, hazard ratios with 95% confidence intervals (CIs) and *p* values (< 0.05) were used to identify statistically significant predictors and to measure the strength of association.

### 2.5. Ethical Considerations

Ethical clearance was obtained from the Institutional Review Board (IRB) of the Jimma University Institute of Health. As the study employed a retrospective design using medical records, the requirement for informed consent was waived by the IRB. No direct patient contact was made, and all data were anonymized prior to analysis. Patient confidentiality was strictly maintained, with only the principal investigator, supervisor, and data collectors having access to the data. The study was conducted in accordance with Good Clinical Practice guidelines and the ethical principles outlined in the Declaration of Helsinki.

## 3. Results

A total of 378 patients were admitted with HF during the study period. Of these, 356 patients (94.2%) with complete medical records were included in the analysis, while the remaining 22 patients (5.8%) were excluded due to incomplete documentation or missing key variables required for the study. The primary survival endpoint was the time to mortality among patients with HF. Among the included patients, 56 (15.7% (95% CI: 12.2%–19.8%)) died, while the remaining patients were censored. The median hospital stay was 11 days (IQR: 7–17), with a range of 1 to 157 days.

### 3.1. Sociodemographic and Clinical Profile

The median (IQR) age of the patients was 55 (38–65) years, with the minimum and maximum ages being 18 and 90 years, respectively. The majority of patients were male, comprising 217 (61%) of the total. Upon admission, the most frequently reported symptoms were shortness of breath (SOB) (96.3%), cough (91.3%), and orthopnea (89.9%). In comparison, palpitations and body weakness were less common, reported by 30.9% and 7% of patients, respectively.

Among the deceased patients, elevated median blood pressure readings were observed: systolic BP at 160 mmHg (IQR: 140–170) and diastolic BP at 100 mmHg (IQR: 71–110). Additionally, a median pulse rate of 120 bpm (IQR: 111–130) indicated tachycardia, a median respiratory rate of 28 breaths per minute (IQR: 26–34) indicated tachypnea, and a median oxygen saturation of 91% (IQR: 85–94) indicated mild hypoxia. Anemia (62.8%) and hypertension (53.3%) were the most frequently observed conditions, while pneumonia was notably prevalent among the deceased patients, affecting 89.3% of them (Table 1).

### 3.2. HF-Specific Data

Ischemic heart disease (IHD) (28.4%) and idiopathic dilated cardiomyopathy (IDCM) (26.7%) were the leading etiological factors among patients admitted with HF. The majority of patients had HF with reduced ejection fraction (HFrEF) (49.2%). Additionally, most patients were categorized as NYHA class III (43.3%) and stage C HF (77.0%). Among the deceased patients, IHD (69.6%), HFrEF (76.8%), NYHA class IV (67.9%), and stage D HF (58.9%) were the most prevalent conditions (Table 2).

### 3.3. Time to Mortality Analysis

**Figure 1:**
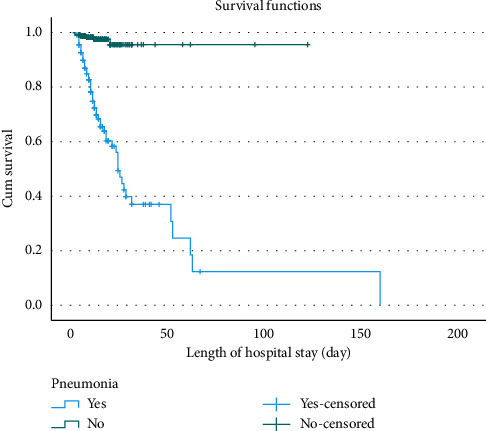
Kaplan–Meier survival curves for patients with heart failure, stratified by pneumonia and duration of hospitalization at Jimma Medical Center, Ethiopia, 2024.

**Figure 2:**
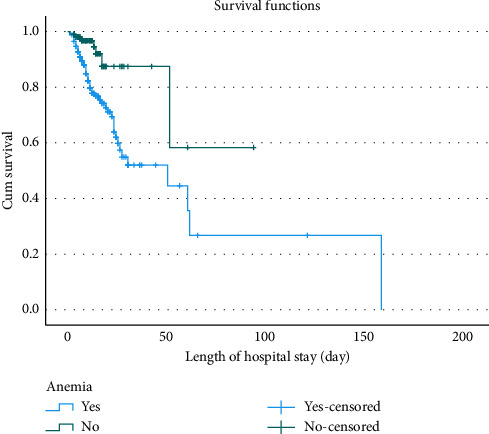
Kaplan–Meier survival curves for patients with heart failure, stratified by anemia and duration of hospitalization at Jimma Medical Center, Ethiopia, 2024.

**Figure 3:**
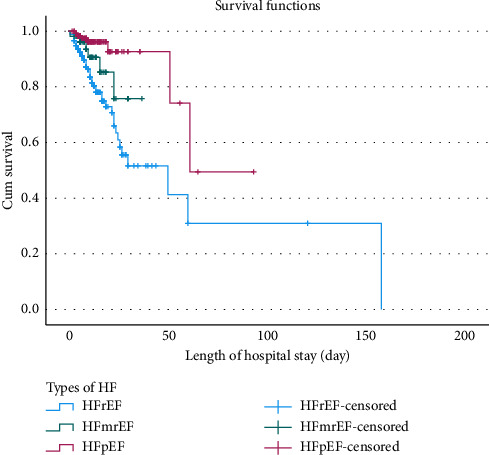
Kaplan–Meier survival curves for patients with heart failure, stratified by type of heart failure and duration of hospitalization at Jimma Medical Center, Ethiopia, 2024.

**Figure 4:**
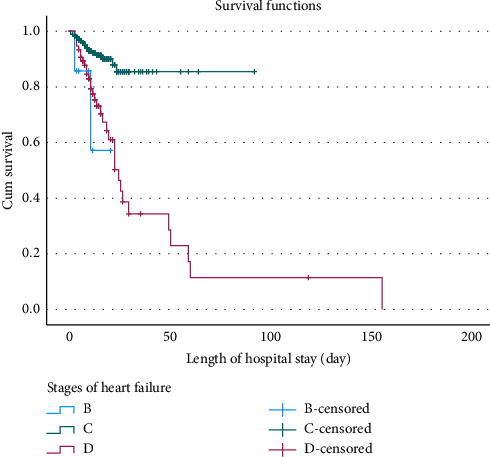
Kaplan–Meier survival curves for patients with heart failure, stratified by stage of heart failure and duration of hospitalization at Jimma Medical Center, Ethiopia, 2024.

The Kaplan–Meier estimator, along with the equality test, was used to estimate survival curves for categorical predictors. Medical conditions such as pneumonia and anemia, as well as the type and stage of HF, were significantly associated with survival outcomes (*p* value < 0.001) (Supporting Table 1). Consequently, patients with HF who had pneumonia (Figure 1), anemia (Figure 2), HFrEF type (Figure 3), or stage D HF (Figure 4) each independently showed a lower probability of survival and required a longer duration of hospital stay.

### 3.4. Predictors of Time to Death

Univariate Cox regression analyses were conducted to assess individual factors associated with the hazard of death among adult HF patients. Variables with fewer than five occurrences were excluded due to insufficient sample size for reliable estimates. Candidate variables for the final multivariate analysis were selected based on their statistical significance in the univariate analyses. Significant factors including medical conditions (hypertension, diabetes mellitus, pneumonia, anemia, acute myocardial infarction [AMI], and hyponatremia), vital signs (SBP, DBP, pulse rate, and respiratory rate), HF etiology (IHD, IDCM, and DCM), and type of HF were then included in the multivariate Cox regression analysis.

Patients admitted with HF who also had other medical conditions, such as hypertension (AHR: 4.6, 95% CI: (1.88–11.61), *p* < 0.001), pneumonia (AHR: 4.3, 95% CI: (1.15–15.78), *p*=0.031), anemia (AHR: 3.3, 95% CI: (1.17–9.06), *p*=0.023), AMI (AHR: 4.4, 95% CI: (1.9–10.09), *p* < 0.001), and hyponatremia (AHR: 2.9, 95% CI: (1.44–5.99), *p*=0.003), faced a higher mortality risk than those without these conditions. Vital signs emerged as significant predictors of mortality among HF patients in our study. Specifically, each unit increase in SBP and DBP was associated with a 7% and 4% lower risk of mortality, respectively. The squared terms for both SBP and DBP, included to explore potential nonlinear relationships, had a significant *p* value of 0.035. This suggests a significant nonlinear effect. Therefore, the data indicate a primarily nonlinear relationship between SBP, DBP, and mortality risk in our study cohort. Additionally, each unit increase in pulse rate was associated with a 4% higher risk of mortality. Furthermore, patients with HFrEF had a six-fold higher risk of mortality (AHR: 6.1, 95% CI: (1.79–24.4), *p*=0.008) compared to those with heart failure with preserved ejection fraction (HFpEF) (Table 3).

## 4. Discussion

In this retrospective cohort study, we examined the time to mortality and predictive factors among adult HF patients admitted to JMC, Ethiopia. Our analysis revealed a notable mortality rate, with significant predictors including medical conditions such as hypertension, pneumonia, anemia, AMI, and hyponatremia, as well as vital signs including SBP, DBP, and pulse rate. Additionally, the type of HF, particularly HF with HFrEF, was identified as a significant predictor of mortality in HF patients.

The median hospital stay of 11 days (IQR: 7–17) in our study is consistent with findings from other studies in LMICs [11–13]. In contrast, developed countries typically report shorter hospital stays, around 5 to 10 days [14]. These differences may reflect variations in healthcare systems, geographic location, and patient characteristics.

The observed mortality rate of 15.7% (95% CI: 12.2%–19.8%) among HF patients in our study is consistent with the 16.5% mortality rate reported in the INTER-CHF study for LMICs, with Africa and India bearing over 50% of the disease burden [13]. This rate is approximately double the mortality observed in high-income countries, where rates typically range from 5% to 10% [15]. The findings highlight the significant disparities in HF outcomes between developing and developed countries. Patients in resource-limited settings face higher mortality risks, likely due to factors such as limited access to guideline-directed medical therapies, delayed diagnoses, and higher prevalence of comorbidities.

In our study, hypertensive patients with HF experienced a 4.6-fold increased risk of mortality compared to those without hypertension. This finding is consistent with most studies [3, 16–19], which indicate that hypertension significantly exacerbates the risk of adverse outcomes in HF patients. This increased risk is due to the compounding effects of hypertension on HF: hypertension imposes additional strain on the heart, leading to accelerated progression of HF [20, 21], worsening of symptoms, and increased susceptibility to complications [22]. The elevated cardiac workload and end-organ damage associated with chronic hypertension further contribute to a higher mortality risk among HF patients [23].

Pneumonia was another medical condition that emerged as a significant predictor of mortality among HF patients in our study. It was associated with a 4.3-fold increase in the hazard of death. This finding aligns with previous studies that have identified pneumonia as a critical comorbidity in HF, contributing to higher mortality rates and worse overall outcomes [3, 24–26]. This elevated risk likely results from the additional strain that pneumonia places on the cardiovascular system [27], further compromising the already weakened heart function in HF patients [24]. Pneumonia exacerbates respiratory demands and reduces oxygenation, leading to acute decompensation in HF [26, 28].

Anemia was another significant predictor of mortality among HF patients in our study, associated with a 3.3-fold increase in the hazard of death. Our finding is consistent with other studies that have highlighted anemia as a common and impactful comorbidity in HF, contributing to increased mortality and poorer clinical outcomes [3, 29–32]. The presence of anemia in HF patients can exacerbate the heart's workload by reducing the oxygen-carrying capacity of the blood [30], leading to tissue hypoxia and further straining the heart [33]. This condition can worsen symptoms of HF, such as fatigue and SOB, and may accelerate the progression of the disease [34].

In our study, AMI was identified as a significant predictor of mortality among HF patients, associated with a 4.4-fold increase in the hazard of death. This finding is consistent with previous research that has identified AMI as a critical event in the clinical course of HF, significantly elevating the risk of mortality and poor outcomes [35–37]. The coexistence of AMI in patients with HF can severely compromise cardiac function, as the infarcted myocardium reduces the heart's ability to pump effectively, leading to further deterioration in HF. AMI can precipitate acute decompensation, increasing the likelihood of fatal arrhythmias and worsening HF symptoms [36, 38].

This study shows that, among patients with HF, presentation with hyponatremia is associated with a 2.9‐fold increase in the hazard of death. These findings are consistent with existing literature that underscores the importance of electrolyte balance in managing HF and its impact on mortality outcomes [39–42]. This elevated risk likely reflects the adverse effects of low sodium levels on cardiovascular and renal function. Hyponatremia often indicates a more severe underlying pathology and contributes to worsening HF by exacerbating fluid imbalance and impairing cardiac function [43–45].

Vital signs emerged as significant predictors of mortality among HF patients in our study. Specifically, each unit increase in SBP was associated with a 7% lower risk of mortality, suggesting that higher SBP may have a protective effect in this population. This finding aligns with some studies that have observed a paradoxical relationship between higher SBP and better outcomes in HF patients [46–50]. Additionally, each unit increase in DBP was associated with a 4% lower risk of mortality, further supporting the notion that maintaining adequate blood pressure may be beneficial in managing HF. These findings support existing literature indicating that higher DBP is often linked with improved outcomes in HF patients [47, 48, 51]. Elevated SBP might indicate better systemic perfusion, helping to maintain adequate blood flow to vital organs and tissues despite impaired cardiac function [48, 52, 53]. Higher DBP, on the other hand, can improve coronary perfusion during diastole, reducing myocardial stress and enhancing cardiac function [54]. This could help offset the effects of HF and contribute to better overall outcomes. Additionally, higher blood pressures might be a marker of preserved cardiac reserve or compensatory mechanisms that temporarily mitigate the severity of HF symptoms [46, 55]. Conversely, each unit increase in pulse rate was associated with a 4% higher risk of mortality, consistent with literature indicating that tachycardia exacerbates the workload on an already compromised heart, leading to poorer outcomes [56–58].

The last significant predictor of mortality among HF patients identified was the type of HF. Specifically, patients with HF with HFrEF had a six-fold higher risk of mortality compared to those with HF with HFpEF. This finding aligns with existing literature, highlighting significant differences in prognosis between these two types of HF [59–61]. The increased mortality in HFrEF likely stems from its association with severe myocardial damage and a more aggressive disease course, leading to higher susceptibility to adverse cardiac events [62, 63].

### 4.1. Strength and Limitation

This study offers valuable insights into the factors influencing mortality among adult HF patients in a resource-limited setting. It enabled a comprehensive analysis of time to mortality and the identification of predictive factors, providing evidence that could inform clinical decision making and management strategies in similar contexts. Additionally, the inclusion of vital signs and comorbid conditions as predictive variables enhances the study's relevance to real-world clinical practice.

However, the retrospective nature of the study presents inherent limitations, including the reliance on the accuracy of medical records, which might introduce information bias. Additionally, excluding patients with missing data could have skewed the results. The study's focus on a single center further limits the generalizability of the findings to other regions or healthcare settings.

## 5. Conclusion

This study reveals crucial predictors of mortality among HF patients at JMC, Ethiopia, where the mortality rate is alarmingly high. Key factors include hypertension, pneumonia, anemia, AMI, hyponatremia, and vital signs like SBP, DBP, and pulse rate. Notably, patients with HFrEF are at significantly higher risk compared to those with HFpEF. These insights call for urgent, targeted interventions. Effective strategies should involve enhancing healthcare policies to improve HF management and comorbidity treatment in resource-limited settings, refining clinical practices with updated guidelines for managing these conditions, and boosting provider training and resources for early detection and comprehensive care. Additionally, further research is needed to understand these predictors better and develop precise interventions. By focusing on these areas, future efforts can significantly reduce mortality rates and elevate care quality for HF patients in similar settings.

## Figures and Tables

**Table 1:** Sociodemographic and clinical profile at admission among adult heart failure patients admitted to Jimma Medical Center, Ethiopia, 2024.

Characteristics	Total (*n* = 356)	Died (*n* = 56)	Survived (*n* = 300)	*p* value
Age, median (IQR)	55 (38–65)	57 (36–65)	55 (38–65)	0.813
Age group				0.620
18–39	91 (25.6)	15 (26.8)	76 (25.3)	
40–64	160 (44.9)	22 (39.3)	138 (46.0)	
≥ 65	105 (29.5)	19 (33.9)	86 (28.7)	
Sex				0.796
Male	217 (61.0)	35 (62.5)	182 (60.7)	
Female	139 (39.0)	21 (37.5)	118 (39.3)	
Residence				0.803
Urban	126 (35.4)	19 (33.9)	107 (35.7)	
Rural	230 (64.6)	37 (66.1)	193 (64.3)	
Symptom profile (yes)				
SOB	343 (96.3)	55 (98.2)	288 (96.0)	0.417
Cough	325 (91.3)	54 (96.4)	271 (90.3)	0.138
Orthopnea	320 (89.9)	53 (94.6)	267 (89.0)	0.199
PND	317 (89.0)	53 (94.6)	264 (88.0)	0.144
Leg swelling	310 (87.1)	52 (92.9)	258 (86.0)	0.160
Chest pain	211 (59.3)	39 (69.6)	172 (57.3)	0.085
Fever	138 (38.8)	26 (46.4)	112 (37.3)	0.200
Body weakness	25 (7.0)	5 (8.9)	20 (6.7)	0.543
Palpitation	110 (30.9)	19 (33.9)	91 (30.3)	0.593
Vital signs, median (IQR)				
SBP (mmHg)	115 (100–140)	160 (140–170)	110 (100–135)	< 0.001^∗^
DBP (mmHg)	70 (60–90)	100 (71–110)	70 (60–85)	< 0.001^∗^
Pulse rate (bpm)	90 (81–100)	120 (111–130)	88 (80–96)	< 0.001^∗^
Respiratory rate (bpm)	26 (24–28)	28 (26–34)	24 (24–28)	< 0.001^∗^
Temperature (°C)	36.6 (36.3–36.8)	36.6 (36.1–37.0)	36.6 (36.3–36.8)	0.991
SpO_2_ ()	94 (90–96)	91 (85–95)	94 (90–96)	0.053
Medical conditions (yes)				
Hypertension	153 (53.3)	46 (82.1)	107 (46.3)	< 0.001^∗^
Diabetes mellitus	55 (19.9)	45 (80.4)	10 (4.5)	< 0.001^∗^
Pneumonia	110 (30.9)	50 (89.3)	60 (20.0)	< 0.001^∗^
COPD	19 (7.4)	4 (8.9)	15 (7.0)	0.667
Asthma	5 (1.9)	3 (6.7)	2 (0.9)	0.011^∗^
Anemia	169 (62.8)	49 (87.5)	120 (56.3)	< 0.001^∗^
CKD	39 (15.1)	9 (20.0)	30 (14.1)	0.314
Stroke	17 (6.6)	4 (8.9)	13 (6.1)	0.494
AMI	69 (22.8)	15 (28.3)	54 (21.6)	0.291
Atrial fibrillation	114 (37.6)	21 (39.6)	93 (37.2)	0.741
Hyponatremia	100 (33.0)	25 (47.2)	75 (30.0)	0.016^∗^
LVT	40 (13.2)	7 (13.2)	33 (13.2)	0.999

*Note:* mmHg: millimeter mercury; °C: degree Celsius; SpO_2_: oxygen saturation.

Abbreviations: AMI, acute myocardial infraction; CKD, chronic kidney disease; COPD, chronic obstructive pulmonary disease; DBP, diastolic blood pressure; LVT, left ventricular thrombus; PND, paroxysmal nocturnal dyspnea; SBP, systolic blood pressure; SOB, shortness of breath.

^∗^ *p* value < 0.05.

**Table 2:** Baseline heart failure–specific profile at admission among adult heart failure patients admitted to Jimma Medical Center, Ethiopia, 2024.

Characteristics	Total (*n* = 356)	Died (*n* = 56)	Survived (*n* = 300)	*p* value
Etiology (yes)				
Ischemic heart disease	101 (28.4)	39 (69.6)	62 (20.7)	< 0.001^∗^
IDCM	95 (26.7)	33 (58.9)	62 (20.7)	< 0.001^∗^
CRVHD	82 (23.0)	15 (26.8)	67 (22.3)	0.468
Dilated cardiomyopathy	72 (20.2)	36 (64.3)	36 (12.0)	< 0.001^∗^
DVHD	17 (4.8)	1 (1.8)	16 (5.3)	0.491
Hypertensive heart disease	11 (3.1)	1 (1.8)	10 (3.3)	1
Infective endocarditis	3 (0.8)	0 (0.0)	3 (1.0)	1
Congenital heart defects	2 (0.6)	0 (0.0)	2 (0.7)	1
Type of heart failure				< 0.001^∗^
HFrEF	175 (49.2)	43 (76.8)	132 (44.0)	
HFmrEF	54 (15.2)	6 (10.7)	48 (16.0)	
HFpEF	127 (35.7)	7 (12.5)	120 (40.0)	
NYHA functional class				< 0.001^∗^
I	1 (0.3)	0 (0.0)	1 (0.3)	
II	74 (20.8)	4 (7.1)	70 (23.3)	
III	154 (43.3)	14 (25.0)	140 (46.7)	
IV	127 (35.7)	38 (67.9)	89 (29.7)	
Stage of heart failure				< 0.001^∗^
B	7 (2.0)	2 (3.6)	5 (1.7)	
C	274 (77.0)	21 (37.5)	253 (84.3)	
D	75 (21.1)	33 (58.9)	42 (14.0)	

*Note:* IDCM: idiopathic dilated cardiomyopathy.

Abbreviations: CRVHD, chronic rheumatic valvular heart disease; DVHD, degenerative valvular heart disease; HFmrEF, heart failure with midrange ejection fraction; HFpEF, heart failure with preserved ejection fraction; HFrEF, heart failure with reduced ejection fraction; NYHA, New York Heart Association.

^∗^ *p* value < 0.05.

**Table 3:** Univariate and multivariate Cox regression analysis of predictors associated with mortality among patients with heart failure admitted to Jimma Medical Center, Ethiopia, 2024.

Variable	CHR (95% CI)	*p* value	AHR (95% CI)	*p* value
Medical conditions				
Hypertension				
Yes	5.1 (2.5–10.55)	< 0.001^∗^	4.6 (1.88–11.61)	0.001^∗^
No	Ref	Ref	Ref	Ref
Diabetes mellitus				
Yes	14.6 (7.5–28.48)	< 0.001^∗^	2.3 (0.89–6.05)	0.1
No	Ref	Ref	Ref	Ref
Pneumonia				
Yes	15.2 (6.5–35.74)	< 0.001^∗^	4.3 (1.15–15.78)	0.031^∗^
No	Ref	Ref	Ref	Ref
Anemia				
Yes	3.43 (1.55–7.6)	0.002^∗^	3.3 (1.17–9.06)	0.023^∗^
No	Ref	Ref	Ref	Ref
AMI				
Yes	2.2 (1.18–4.07)	0.013^∗^	4.4 (1.9–10.09)	< 0.001^∗^
No	Ref	Ref	Ref	Ref
Hyponatremia				
Yes	1.35 (1.05–2.34)	0.002^∗^	2.9 (1.44–5.99)	0.003^∗^
No	Ref	Ref	Ref	Ref
Vital signs				
SBP	1.03 (1.02–1.04)	< 0.001^∗^	0.93 (0.81–0.98)	< 0.001^∗^
DBP	1.04 (1.02–1.05)	< 0.001^∗^	0.96 (0.94–0.98)	0.001^∗^
Pulse rate	1.04 (1.03–1.05)	< 0.001^∗^	1.04 (1.02–1.06)	< 0.001^∗^
Respiratory rate	1.04 (1.03–1.047)	< 0.001^∗^	0.98 (0.93–1.05)	0.824
Etiology				
IHD				
Yes	7.4 (4.16–13.15)	< 0.001^∗^	1.03 (0.42–2.59)	0.945
No	Ref	Ref	Ref	Ref
IDCM				
Yes	4.2 (2.45–7.2)	< 0.001^∗^	0.7 (0.31–1.47)	0.317
No	Ref	Ref	Ref	Ref
DCM				
Yes	5.6 (3.22–9.77)	< 0.001^∗^	1.9 (0.72–5.43)	0.296
No	Ref	Ref	Ref	Ref
Type of HF				
HFrEF	4.4 (2–9.9)	< 0.001^∗^	6.1 (1.79–24.4)	0.008^∗^
HFmrEF	2.2 (0.76–6.8)	0.143	2.7 (0.63–11.5)	0.178
HFpEF	Ref	Ref	Ref	Ref

*Note:* Ref, reference; IDCM: idiopathic dilated cardiomyopathy; DCM; dilated cardiomyopathy.

Abbreviations: AHR, adjusted hazard ratio; AMI, acute myocardial infraction; CHR, crude hazard ratio; CI, confidence interval; DBP, diastolic blood pressure; HFmrEF, heart failure with midrange ejection fraction; HFpEF, heart failure with preserved ejection fraction; HFrEF, heart failure with reduced ejection fraction; IHD, ischemic heart disease; SBP, systolic blood pressure.

^∗^Indicated a significance association (*p* value of < 0.05).

## Data Availability

All relevant data are within the manuscript.

## Nomenclature

AHR: Adjusted hazard ratio
AMI: Acute myocardial infarction
CHR: Crude hazard ratio
CIs: Confidence intervals
CKD: Chronic kidney disease
COPD: Chronic obstructive pulmonary disease
CRVHD: Chronic rheumatic valvular heart disease
DBP: Diastolic blood pressure
DVHD: Degenerative valvular heart disease
ESCP: Ethiopian Society of Cardiac Professionals
HF: Heart failure
HFrEF: Heart failure with reduced ejection fraction
HFmrEF: Heart failure with midrange ejection fraction
HFpEF: Heart failure with preserved ejection fraction
IQR: Interquartile range
JMC: Jimma Medical Center
LMICs: Low- and middle-income countries
LVT: Left ventricular thrombus
NYHA: New York Heart Association
PND: Paroxysmal nocturnal dyspnea
SOB: Shortness of breath
SBP: Systolic blood pressure
SPSS: Statistical Package for the Social Sciences
SpO_2_: Oxygen saturation
°C: Degrees Celsius
mmHg: Millimeters of mercury

## Supporting Information

Supporting table 1: equality test of survival distributions.
