# Supplementary material for: Time to Mortality and Predictive Factors Among Adult Heart Failure Patients: Lessons From a Resource-Limited Setting
Source: Cardiol Res Pract. 2025 Aug 7;2025:3968055. doi: 10.1155/crp/3968055 (PMC12352995; doi:10.1155/crp/3968055)
Supplement: Supporting Information — Additional supporting information can be found online in the Supporting Information section. [file 3968055.f1.docx]

| Tests | Pneumonia | | Anemia | | Types of HF | | Stages of HF | |
| --- | --- | --- | --- | --- | --- | --- | --- | --- |
|  | Chi-Square | P-value | Chi-Square | P-value | Chi-Square | P-value | Chi-Square | P-value |
| Log Rank (Mantel-Cox) | 69.406 | <0.001* | 10.49 | 0.001* | 17.102 | <0.001* | 33.15 | <0.001* |
| Breslow (Generalized Wilcoxon) | 54.525 | <0.001* | 9.805 | 0.002* | 13.727 | 0.001* | 16.661 | <0.001* |
| Tarone-Ware | 64.35 | <0.001* | 10.644 | 0.001* | 16.273 | <0.001* | 23.312 | <0.001* |

*Indicates p value ≤0.005
